# Cardiopulmonary fitness predicts postoperative major morbidity after esophagectomy for patients with cancer

**DOI:** 10.14814/phy2.14174

**Published:** 2019-07-24

**Authors:** Neil Patel, Arfon G. Powell, Jenni R. Wheat, Christopher Brown, Ian R. Appadurai, Richard G. Davies, Damian M. Bailey, Wyn G. Lewis

**Affiliations:** ^1^ Department of General Surgery University Hospital of Wales Cardiff United Kingdom; ^2^ Division of Cancer and Genetics Cardiff University School of Medicine Heath Park Cardiff United Kingdom; ^3^ Department of Anaesthetics University Hospital of Wales Cardiff United Kingdom; ^4^ Neurovascular Research Laboratory, Faculty of Life Sciences and Education University of South Wales Pontypridd United Kingdom

**Keywords:** Cardiopulmonary fitness, morbidity, esophageal cancer, prognosis

## Abstract

Surgery for radical treatment of esophageal cancer (EC) carries significant inherent risk. The objective identification of patients who are at high risk of complications is of importance. In this study the prognostic value of cardiopulmonary fitness variables (CPF) derived from cardiopulmonary exercise testing (CPET) was assessed in patients undergoing potentially curative surgery for EC within an enhanced recovery program. OC patients underwent preoperative CPET using automated breath‐by‐breath respiratory gas analysis, with measurements taken during a ramped exercise test on a bicycle. The prognostic value of $\dot{V}{\text{O}}_{2\text{Peak}}$, Anaerobic Threshold (AT) and VE/VCO_2_ derived from CPET were studied in relation to post‐operative morbidity, which was collected prospectively, and overall survival. Consecutive 120 patients were included for analysis (median age 65 years, 100 male, 75 neoadjuvant therapy). Median AT in the cohort developing major morbidity (Clavien–Dindo classification >2) was 10.4 mL/kg/min compared with 11.3 mL/kg/min with no major morbidity (*P* = 0.048). Median $\dot{V}{\text{O}}_{2\text{Peak}}$ in the cohort developing major morbidity was 17.0 mL/kg/min compared with 18.7 mL/kg/min in the cohort (*P* = 0.009). $\dot{V}{\text{O}}_{2\text{Peak}}$ optimum cut‐off was 17.0 mL/kg/min (sensitivity 70%, specificity 53%) and for AT was 10.5 mL/kg/min (sensitivity 60%, specificity 44%). Multivariable analysis revealed $\dot{V}{\text{O}}_{2\text{Peak}}$ to be the only independent factor to predict major morbidity (OR 0.85, 95% CI 0.75–0.97, *P* = 0.018). Cumulative survival was associated with operative morbidity severity (χ^2^ = 4.892, df = 1, *P* = 0.027). These results indicate that $\dot{V}{\text{O}}_{2\text{Peak}}$ as derived from CPET is a significant predictor of major morbidity after oesophagectomy highlighting the physiological importance of cardiopulmonary fitness.

## Introduction

Oesophagectomy remains the primary therapeutic modality for radical and potentially curative treatment for patients with esophageal cancer (EC), but despite recent advances in anesthesiology and critical care, it continues to carry significant inherent risk. Indeed, the 2016 UK National Oesophago‐Gastric Cancer Audit (National Oesophago‐Gastric Cancer Audit, 2018) reported postoperative morbidity and mortality of 50% and 1.6% respectively.

Comprehensive and accurate risk profile assessment encompassing preoperative modifiable factors and aerobic capacity should be fundamental within the decision‐making process for the multidisciplinary team, and patient, when selecting the most appropriate management modality, particularly in patients who are elderly and with comorbidities. Moreover, such a strategy permits patient optimization prior to surgery and efficient utilization of critical care resources. Current approaches to risk prediction comprise: clinical acumen, objective scoring systems such as the Portsmouth Physiological and Operative Severity Score for the Enumeration of Mortality and Morbidity (P‐POSSUM) (Whiteley et al. 1996), Oesophagogastric POSSUM (O‐POSSUM) (Tekkis et al. 2004), American Society of Anesthesiologists (ASA) physical status, Charleston Comorbidity Index, plasma biomarkers, measures of cardiac function (Moyes et al. 2013) and shuttle walk tests (Murray et al. 2007). Their effectiveness in predicting surgical morbidity is relatively weak and measures to improve this process are needed.

Cardiopulmonary exercise testing (CPET) is a dynamic and noninvasive procedure, which allows approximation of an individual’s cardiopulmonary fitness to be measured. This results in an objective assessment of ability to cope with the metabolic demands associated with the physiological trauma of major surgery (Smith et al. 2009). CPET, in particular an anaerobic threshold <11 mL/kg/min, has been shown to predict post‐operative morbidity and mortality in patients undergoing major abdominal surgery (Older et al. 1993; Snowden et al. 2013; Moran et al. 2016). Although CPET is well established in cardiothoracic surgery (American Thoracic Society and American College of Chest Physicians, 2003), the number of studies assessing the value of CPET related to oesophagectomy is few Moran et al. 2016, and none have assessed its value within the framework of an enhanced recovery program (ERP). Enhanced recovery programs, long embedded with gastrointestinal cancer surgical practice, have been associated with reduced postoperative morbidity and shortened duration of hospital stay (Fearon et al. 2005; Karran et al. 2016). The primary aim of this study was to define the baseline cardiopulmonary fitness levels of patients undergoing radical surgical treatment in an ERP and to establish critical threshold values that identify patients at increased risk of developing major postoperative morbidity as stratified by the Clavien–Dindo Classification (Dindo et al. 2004). Clavien–Dindo classification is an ordinal measure of the severity of postoperative morbidity, which is reproducible and applies to all patients undergoing surgical procedures. The hypothesis was that cardiopulmonary fitness, namely $\dot{V}{\text{O}}_{2\text{Peak}}$ and anaerobic threshold (AT), would be inversely associated with major postoperative morbidity in patients undergoing oesophagectomy. The setting was a regional Upper Gastrointestinal cancer network multidiciplinary team (MDT) serving a population of 1.8 million.

## Methods

### Study approval

The sponsor of this study was C&V UHB General Surgery Directorate who deemed it to be in keeping with audit and service evaluation. Audit and service evaluation is part of quality assurance. These involve minimal additional risk, burden or intrusion for participants, are regulated outside of the Research Ethics system, and do not require approval from the NHS Research and Development offices. This opinion was confirmed and approved by Health and Care Research Wales Research Ethics Service.

### Study design

Consecutive patients diagnosed with esophageal (including oesophagogastric junctional type 1 and 2) cancer of any cell type, between August 2010 and August 2016, considered for surgical treatment by an MDT were identified and referred for prospective CPET testing. Patients undergoing total gastrectomy for junctional type 3 cancers and palliative procedures were excluded.

### Patients

Data relating to the preoperative status, operative procedure and outcome were collected prospectively. The preoperative assessment process was defined as the process from diagnosis to the time of anesthesia for definitive surgery. This period also included the completion of the radiological staging process. Data which were collected included age, gender, American Association of Anesthesiology (ASA) grade, radiological and histopathological stage of disease (TNM7) (Sobin et al. 2009), cancer site, operative mortality (defined in this study as 30 day or inpatient death on index admission), operative morbidity graded in accordance with the Clavien–Dindo Classification (CDC) (Dindo et al. 2004), duration or length of hospital stay (LOHS), and survival from date of diagnosis.

### Surgical treatment and neoadjuvant therapy

All patients had management plans individually tailored according to factors relating to both the patient and their disease. Staging was by means of computed tomography, endoscopic ultrasound, computed tomography positron emission tomography (CT PET) and staging laparoscopy as appropriate. The South East Wales MDT treatment algorithms for EC have been described previously (Morgan et al. 2009). The standard operation consisted of subtotal transthoracic oesophagectomy (TTO) as described by Lewis (1946) and Tanner (1947). Transhiatal oesophagectomy (THO), as described by Orringer (1985), was used selectively in patients with adenocarcinoma of the lower third of the esophagus who had significant cardiorespiratory comorbidity, T1/2 N0 or T3 N0 disease. All procedures were performed using an open approach.

### CPET testing

The CPET followed American Thoracic Society/American College of Chest Physicians recommendations (American Thoracic Society and American College of Chest Physicians, 2003), and performed prior to any treatment commencing. All patients performed a symptom limited CPET conducted on an electromagnetically braked cycle ergometer, and comprised a 2–3 min rest phase (to allow gas exchange variables to stabilize), 3 min unloaded cycling, then a ramped incremental protocol until volitional termination, and a 2–min recovery period. Ventilation and gas exchange was measured with a Medgraphics Ultima^TM^ metabolic cart (Medical Graphics, St Paul, Minnesota) with Breezesuite^TM^ and Welch Allyn^®^ (Welch Allyn, Inc., NY) software. Metabolic data were collected breath‐by‐breath through a mouthpiece with saliva trap using mid five of seven breath averaging. Resting spirometry was performed prior to each exercise test (Rose et al. 2018; Rose et al. 2018).

Heart rate, full 12‐lead electrocardiogram, blood pressure and pulse oximetry were monitored throughout. The ramp gradient was set to 10–20 Watts based on the predicted $\dot{V}{\text{O}}_{2\text{Peak}}$ from the age, weight, height and sex of the patient in order to produce an exercise test of between 8–12 min duration (Wasserman, 2012). Prior to each test, the CPET equipment was calibrated against reference gases. The AT was determined using the V‐slope method and confirmed by changes in ventilatory efficiency for oxygen (VE/V0_2_) and end‐tidal partial pressure values for oxygen (PET_O2_) (Wasserman, 2012). AT was validated independently by two experienced clinicians (IA and RD). $\dot{V}{\text{O}}_{2\text{Peak}}$ was the highest $\dot{V}{\text{O}}_{2}$ achieved during the final 30 sec of the test. The VE/VCO_2_ slope was measured at the AT. Test termination criteria included: request of patient, volitional fatigue, chest or leg pain, or electrocardiographic abnormalities determined by the consultant anesthetist.

### Enhanced recovery program

An ERP was integral to the surgical model based on the principles introduced by Basse and colleagues (Basse et al. 2000). Multimodal programs (transthoracic and transhiatal) were developed following an information gathering process inclusive of surgical, oncological, radiological, dietetic, nursing, and physiotherapy staff, including a literature review to inform specific pathway aspects (Karran et al. 2014). Pathway booklets were created, which served as a unified multidisciplinary patient record, within which all documentation was centralized. The standardized anesthetics approach comprised a thoracic epidural followed by a general anesthetic. Arterial and central venous lines were used in all patients and goal directed fluid therapy was utilized with the aid of lithium dilution cardiac output monitoring (LiDCO Ltd. Copyright © LiDCO 2015. Company registered in England No. 2659005).

### Outcome measures

Primary outcome measures were major operative morbidity, related to CDC severity grade (Dindo et al. 2004), 30‐day operative mortality, and cumulative overall survival in months from date of diagnosis. Particular emphasis was placed on the incidence of morbidity of CDC grade III or higher, as this represented a complication requiring endoscopic, radiological or surgical intervention. A secondary outcome measure was duration or length of hospital stay (LOHS) in days. No patients were lost to follow‐up, and dates and causes of death were obtained by the Wales Cancer Intelligence and Surveillance Unit, from the Office of National Statistics. Patients were followed‐up at 3‐monthly intervals for the first year, then 6‐monthly for the second year and then annually thereafter for a minimum of 5 years or death. Patients underwent clinical and blood evaluation, including Carcino Embryonic Antigen (CEA) measurement, at each clinic appointment. Patients who presented with symptoms suggestive of recurrent disease underwent a CT scan of the thorax, abdomen and pelvis, supplemented with endoscopic evaluation if indicated. The proportion of patients followed‐up for 1, 3 and 5 years was 108 (90.0%), 71 (59.2%) and 42 (35.0%) respectively.

### Statistical analysis

Statistical analysis was performed using SPSS^®^ (IBM^®^ SPSS^®^ Statistics v20.0.0.2, IBM Corporation, Armonk, New York). Shapiro–Wilk tests were used to assess distribution normality. Grouped data were expressed as median (range) and nonparametric analyses were used throughout. Results were considered to be statistically significant at the 5% level. Categorical data were compared using the chi‐squared test, except where groups contained counts of fewer than five, when Fisher’s exact test was used. Grouped continuous data were compared using the Mann–Whitney *U*‐test. Non‐parametric Receiver Operator Characteristic (ROC) curves were used for the predictive value of CPET variables (Youden, 1950). Logistic regression was used to determine the association of CPET variables with morbidity. Variables associated with major morbidity on univariable analysis at the *P* < 0.10 level were entered into a multivariate binary logistic regression analysis, using a forward conditional stepwise method. LOHS and survival analyses were conducted using the conventional method described by Kaplan and Meier.

## Results

During the study period, 180 patients underwent CPET. Of these, 60 patients did not proceed to resection and were excluded from further analysis (23 deemed unfit, 25 had cancer stage progression, and 12 had open and close procedures). Therefore, 120 patients (100 males, 20 females) were included in the analysis. There were no differences in age (*P* = 0.893), gender (*P* = 0.926), or ASA grade (*P* = 0.463), between the no major morbidity and major morbidity cohorts. More THOs were performed (76, 63.3%) compared with TTO procedures (44, 36.7%). Major morbidity was similar after both operative approaches (THO 25.0% vs. TTO 27.3%, *P* = 0.784) (Table 1). Seventy‐five patients received neoadjuvant therapy as part of the NEOSCOPE trial (Mukherjee et al. 2015): 60 receiving chemotherapy and 15 chemoradiotherapy.

**Table 1:** Patient demographics, combined radiological and histopathological stage related to Clavien–Dindo grade ≥3 morbidity.

	Total	No Major Morbidity	Major Morbidity	*P*‐value
	*n* = 120	*n* = 89	*n* = 31	
Age (years)^1^	65 (38–84)	65 (42–84)	65 (38–75)	0.893
Gender (M:F)	100:20	74:15	26:5	0.926
ASA grade				0.463
I	9 (7.5)	8 (8.9)	1 (3.2)	
II	72 (60.0)	54 (60.6)	18 (58.1)	
III	39 (32.5)	27 (30.3)	12 (38.7)	
CPET variables				
AT (mL/kg/min)	11.0 (7.0–22.3)	11.3 (7.0–22.3)	10.4 (7.6–15.9)	0.048
$\dot{V}{\text{O}}_{2\text{Peak}}$ (mL/kg/min)	18.2 (10.9–38.9)	18.7 (11.3–38.8)	16.9 (10.9–26.2)	0.009
VE/VCO_2_	30.0 (13.3–44.1)	30.0 (21.5–44.1)	32.0 (13.3–39.2)	0.584
Neoadjuvant therapy				0.964
None	45 (37.5)	34 (38.2)	11 (35.5)	
Chemotherapy	60 (50.0)	44 (49.4)	16 (51.6)	
Chemo radiotherapy	15 (12.5)	11 (12.4)	4 (12.9)	
Surgery				0.784
Transthoracic	44 (36.7)	32 (36.0)	12 (38.7)	
Transhiatal	76 (63.3)	57 (64.0)	19 (61.3)	
Radiological T Stage				0.870
Tx	4 (3.3)	3 (3.4)	1 (3.3)	
T1	21 (17.5)	15 (16.9)	6 (19.4)	
T2	17 (14.2)	14 (15.7)	3 (9.7)	
T3	70 (58.3)	52 (58.4)	18 (58.1)	
T4	8 (6.7)	5 (5.6)	4 (9.7)	
Radiological N Stage				0.962
N0	75 (62.5)	56 (62.9)	19 (61.3)	
N1	32 (26.7)	24 (27.0)	8 (25.8)	
N2	9 (7.5)	6 (6.7)	3 (9.7)	
N3	4 (3.3)	3 (3.4)	1 (3.3)	
Pathological T Stage				0.005
HGD^2^	5 (4.2)	2 (2.2)	3 (9.7)	
T1	28 (23.3)	26 (29.2)	2 (6.5)	
T2	19 (15.8)	12 (13.5)	7 (22.6)	
T3	62 (51.7)	47 (52.8)	15 (48.4)	
T4	6 (5.0)	2 (2.2)	4 (12.9)	
Pathological N Stage				0.088
N0	58 (48.3)	46 (51.7)	12 (38.7)	
N1	32 (26.7)	19 (21.3)	13 (41.9)	
N2	24 (20.0)	18 (20.2)	6 (19.4)	
N3	6 (5.0)	6 (6.7)	0 (0.0)	
Resection Margin				0.740
R0^3^	65 (54.2)	49 (55.1)	16 (51.6)	
R1	55 (45.8)	40 (44.9)	15 (48.4)	

Values in parentheses are percentages; ^1^Values are median (range); AT, anaerobic threshold; $\dot{V}{\text{O}}_{2\text{Peak}}$, oxygen uptake at peak exercise; VE/VCO_2_, minute ventilation; ^2^High Grade Dysplasia; ^3^R0, Resection margin clear; R1 resection margin involved (microscopic circumferential resection margin positive).

### Major morbidity

Seventy‐seven patients (64.2%) developed postoperative morbidity of any CDC grade and 31 patients (25.8%) experienced major morbidity (CDC grade ≥ III). The major morbidity included: 12 patients (10.0%) who developed an anastomotic leak with seven requiring operative intervention; 11 (9.2%) developed significant respiratory complications (1 ARDS, 2 pneumothorax, eight chest sepsis), 2 (1.6%) developed chyle leaks (both managed operatively), 2 (1.6%) developed postoperative bleeding requiring operative intervention, 2 (1.6%) developed small bowel ischemia, one developed multi organ failure, and one patient developed a bile leak following pyloroplasty. Of the 31 patients developing major morbidity, 20 (64.5%) received neoadjuvant therapy (16 chemotherapy and four chemoradiotherapy) and 11 (35.5%) underwent surgery alone, *P* = 0.788 (Table 1). There were four deaths (3.3%) within 30 days of surgery and the causes of death were: anastomotic leak (two patients), Acute Respiratory Distress Syndrome (1), and secondary intrathoracic hemorrhage (1).

Four patients had indeterminate AT (three respiratory exchange ratio (RER) >1, and one patient was unable to complete the test); all developed major morbidity (Table 2). For the complete cohort the baseline median AT was 11.0 mL/kg/min (range 7.0–22.3), median $\dot{V}{\text{O}}_{2\text{Peak}}$ was 18.2 mL/kg/min (range 10.9–38.76), and median VO/VCO_2_ was 30.0 (range 13.3–44.0).

**Table 2:** Univariable and multivariable analysis of preoperative factors associated with major morbidity.

	Univariable	*P*‐value	Multivariable	*P*‐value
	Odds ratio (95% CI)		Odds ratio (95% CI)	
Age (years) (<65/66–75/>75)	0.87 (0.34–2.19)	0.761		
Gender (Male/Female)	1.65 (0.46–5.89)	0.441		
ASA (I/II/III)	1.10 (0.47–2.55)	0.827		
Operative approach (Transhiatal/Transthoracic)	0.89 (0.53–1.49)	0.648		
Neoadjuvant therapy (Yes/No)	0.78 (0.22–2.77)	0.695		
Radiological T stage (X/1/2/3/4)	0.91 (0.49–1.72)	0.775		
Radiological N stage (0/1/2/3)	1.00 (0.51–2.00)	0.991		
AT (mL/kg/min)	0.81 (0.66–1.00)	0.053		0.537
$\dot{V}{\text{O}}_{2\text{Peak}}$ (mL/kg/min)	0.85 (0.75–0.97)	0.014	0.85 (0.75–0.97)	0.018
VE/VCO_2_	0.98 (0.88–1.08)	0.640		

**Figure 1:**
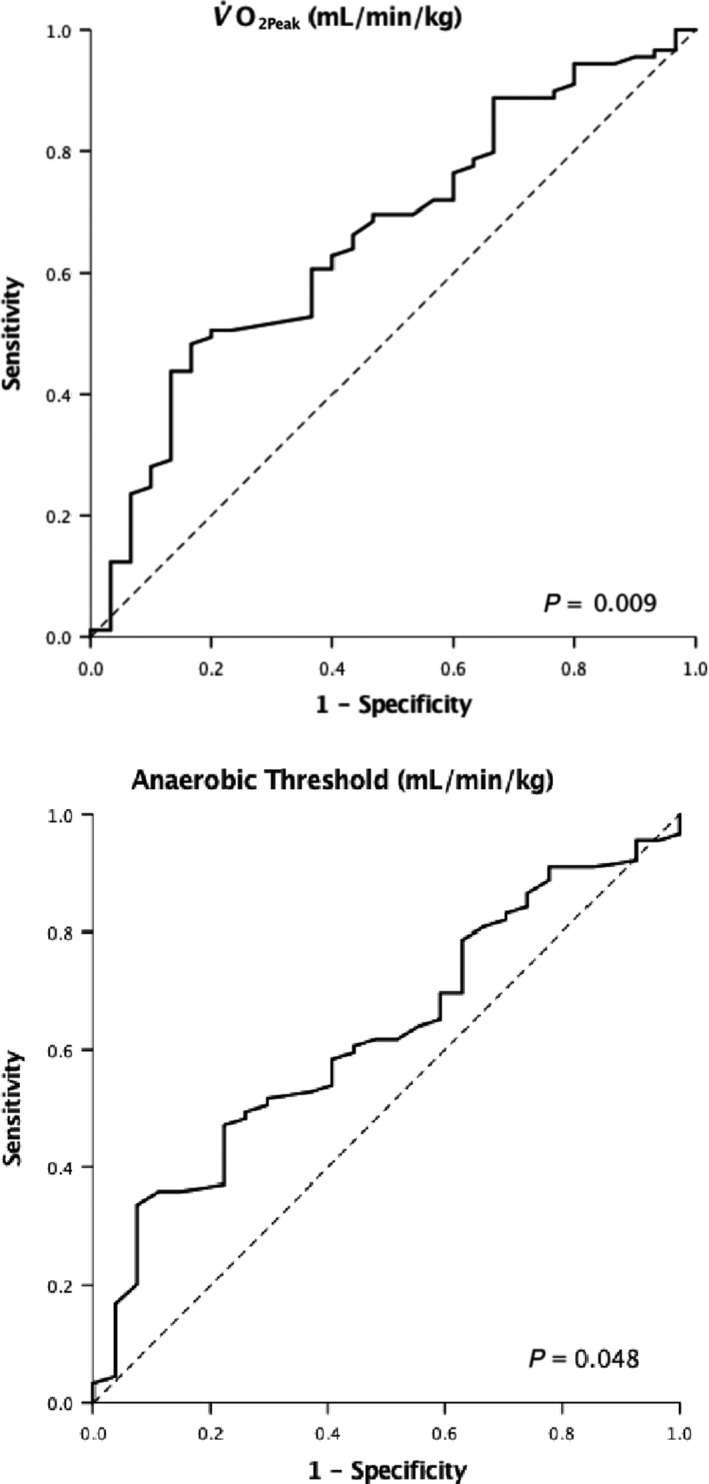
Cardiopulmonary fitness variable ROC curves.

Table 3 shows cardiopulmonary fitness variables related to operative approach. Nonparametric ROC curves illustrated that both $\dot{V}{\text{O}}_{2\text{Peak}}$ and AT were associated with major morbidity (Fig. 1). For $\dot{V}{\text{O}}_{2\text{Peak}}$ (area under the curve 0.66, 95% CI 0.55–0.77, *P* = 0.009), the optimal cut‐off point was 17.0 mL/kg/min, giving sensitivity of 70% and specificity of 53%.

**Table 3:** Cardiopulmonary exercise variables related to operative approach.

Variable	THO	TTO	*P*‐value
AT (mL/min/kg)	10.7 (7.0–22.3)	11.9 (7.0–16.8)	0.188
$\dot{V}{\text{O}}_{2\text{Peak}}$ (mL/min/kg)	17.9 (11.9–38.8)	18.4 (10.9–25.9)	0.219
VE/VCO_2_	30.0 (13.3–44.0)	30.0 (23.0–40.0)	0.771

Numbers are median (range).

For AT (area under the curve 0.62, 95% CI 0.51–0.74, *P* = 0.048) the optimal cut‐off point was 10.5 mL per kg per min, giving sensitivity of 60% and specificity of 44%. Patients with a $\dot{V}{\text{O}}_{2\text{Peak}}$ lower than 17.0 mL/kg/min were twice as likely to develop major morbidity than patients with a higher $\dot{V}{\text{O}}_{2\text{Peak}}$, 38.6% versus 18.4% respectively, *P* = 0.015. Patients with AT lower than 10.5 mL/kg/min were twice as likely to develop major morbidity than patients with higher AT, 35% versus 18% respectively, *P* = 0.034. Multivariable analysis, Table 2, revealed $\dot{V}{\text{O}}_{2\text{Peak}}$ to be the only factor independently associated with morbidity of CDC grade ≥ III (OR 0.85, 95% CI 0.75–0.97, *P* = 0.018).

### Duration of hospital stay

The overall median duration or length (LOHS) for all patients was 16 (9–153) days. Patients categorized with morbidity severity scores of CDC grade 0, I and II had a median LOHS of 15 (10–49) days, compared with a LOHS of 35 (9–153) days in patients categorized with morbidity severity scores of CDC grade ≥ III (*P* < 0.001). The LOHS was, on average, one day shorter in patients with $\dot{V}{\text{O}}_{2\text{Peak}}$ values above the cut‐off value than patients below the cut‐off value, median 16 (9–106) versus 15 (9–153) days, *P* = 0.040. No difference in LOHS was observed when dichotomized above and below the AT cut‐off value of 10.5 mL/kg/min, with both cohorts’ LOHS equating to 16 days, *P* = 0.273.

### Survival

**Figure 2:**
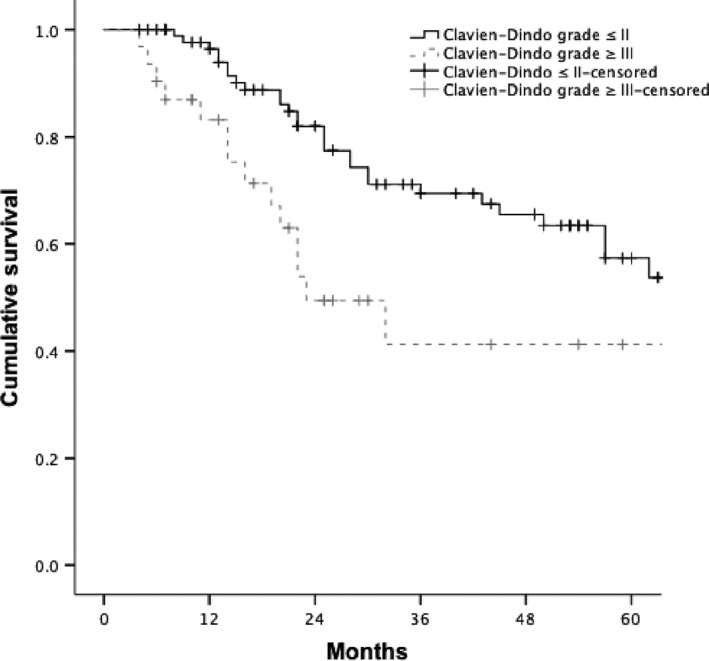
Cumulative overall survival related to major morbidity after oesophagectomy.

The overall median cumulative survival for all patients was 64 (4–85) months. There were 44 deaths, of which four were postoperative deaths, and were included in the survival analysis. The 1‐, 3‐ and 5‐year overall survival rates were 93.51%, 64.78% and 54.76% respectively. Overall survival was not associated with any CPET variables (supplementary Table S1). Not receiving neoadjuvant therapy (*P* = 0.012), higher radiological (r) T stage (*P* = 0.001), rN stage (*P* = 0.006), higher pathological (p) T stage (*P* < 0.001), pN stage (*P* < 0.001), circumferential resection margin positivity (*P* < 0.001), and high CDC grade (*P* = 0.031, Fig. 2), were associated with poor overall cumulative survival. On multivariable analysis higher rN stage (HR 1.47 95% CI 1.02–2.13, *P* = 0.041), pN stage (HR 1.61 95% CI 1.14–2.26, *P* = 0.006), CRM positivity (HR 2.57 95% CI 1.27–5.19, *P* = 0.008), and CDC morbidity (HR 2.60 95% CI 1.32–5.14, *P* = 0.006), were independently associated with poor overall cumulative survival (supplementary Table S1). OM 1‐, 3‐ and 5‐year survival was 81.5%, 62.5% and 42.9% versus 97.5%, 70.7% and 62.5% for the cohort without OM.

## Discussion

This study represents the largest contemporary report regarding the value of CPET in the risk profile assessment of consecutive patients diagnosed with EC, and suitable for potentially curative radical multimodal ERP enhanced treatment, in a regional high volume Upper Gastro Intestinal (UGI) cancer network. The principal findings were that CPET was an objective risk assessment tool before oesophagectomy, and the best prognostic markers of major postoperative morbidity were AT (optimal cut‐off 10.5 mL per kg per min), and $\dot{V}{\text{O}}_{2\text{Peak}}$ (optimal cut‐off of 17.0 mL per kg per min), similar to values reported and related to major abdominal surgery (Older et al. 1993; Older et al. 1999), pancreatic (Ausania et al. 2012; Chandrabalan et al. 2013), colorectal (West et al. 2014; West et al. 2014) and bariatric surgery (Moran et al. 2016). Patients with poorer $\dot{V}{\text{O}}_{2\text{Peak}}$ and AT values below these critical levels were over twice as likely to develop major morbidity than patients with values above the critical levels, and $\dot{V}{\text{O}}_{2\text{Peak}}$ was independently associated with postoperative major morbidity, which consequently had an adverse influence on long‐term survival which was 1.5 times better in the absence of major operative morbidity (OM 5‐year survival 97.5% vs. 62.5% for cohort without OM).

This study extends literature supporting objective measures of physical fitness derived by CPET for risk assessment in major abdomino‐thoracic surgery (Ross et al. 2016). Nagamatsu et al from Kitakyushu City, Japan, reported that cardiopulmonary complications were associated with maximum oxygen uptake for patients having oesophagectomy with a three‐field lymphadenectomy, with surgery performed safely on patients with a maximum oxygen uptake of at least 800 mL per min per m^2^ (Nagamatsu et al. 2001). Forshaw et al from St Thomas’ hospital, London, England, reported $\dot{V}{\text{O}}_{2\text{Peak}}$ to be significantly poorer in patients developing cardiopulmonary complications after oesophagectomy (19.2 mL/min/kg in the complication cohort and 21.4 mL/min/kg in the no complication cohort), allied with a trend towards poorer ATs, and concluded that CPET was of limited value in predicting postoperative cardiopulmonary morbidity (Forshaw et al. 2008). However, these reports described cardiopulmonary complications alone, observed after surgery performed outside the framework of an ERP, in contrast to all major morbidity (graded by severity) within an established ERP. Whether single variable endpoints derived from CPET are associated with cumulative survival remains uncertain, but in the present study, all of the patients who died within one year of surgery, seven (5.8%) had a $\dot{V}{\text{O}}_{2\text{Peak}}$ of <21.2 mL per kg per min, and developed postoperative in‐hospital morbidity, which may arguably represent an endpoint related to survival in such patient cohorts.

No differences were observed in preoperative physical fitness or postoperative morbidity between patients receiving neoadjuvant chemotherapy or chemoradiotherapy, when compared with patients proceeding to surgery alone. Recently, it has been reported that preoperative chemotherapy may cause physical deconditioning and impair cardiopulmonary function (Jack et al. 2014; Sinclair et al. 2016). Neither the impact of neoadjuvant chemotherapy or chemoradiotherapy on postoperative morbidity, nor the benefits of improving preoperative fitness by means of exercise interventions have yet to be established.

The strengths of this study include the consecutive nature of patient assessment for eligibility, the homogeneous study population, the use of the Clavien–Dindo morbidity severity score, the clinical management by an established and experienced multidisciplinary team whose results are well audited and stand up to international comparison (Karran et al. 2016), and the fact that all patients received the benefit of an ERP (Karran et al. 2016). Moreover, the survival data is particularly robust as none of the patients were lost to follow‐up. Potential limitations include the single center design, which limits the generalizability of the data, as well as the ROC curve critical levels that were optimized and derived for this local patient cohort as part of service evaluation. The numbers of patients in this contemporary cohort is modest and the possibility exists that critical CPET values for some outcomes have failed to emerge because of the possible influence of selection bias, type II statistical error, or both. Moreover, AT has been shown to be subject to biological and analytical variation which may influence threshold values for clinical decision making (Davison et al. 2012). The number of operative deaths, four, is also too few to draw any meaningful conclusion on the influence of CPET variables on mortality prediction. The median ATs observed in all levels of morbidity severity was lower than that reported widely in the literature and associated with poor physical fitness, although no internationally agreed critical cut‐off value for AT exists. This implies that patient selection for surgery was not influenced solely by this variable. Such poor physiological fitness may arguably be due to multiple factors such as; deprivation, malnutrition, and advanced stage of disease. Clearly this was not a randomized controlled trial and so no comparison group exists to compare the impact of CPET on patient outcomes.

### Clinical implications

Mostly, AT has been regarded as the principal exercise variable for identifying high‐risk patients undergoing surgery. Similar to Forshaw et al, this study found $\dot{V}{\text{O}}_{2\text{Peak}}$ to be a statistically stronger predictor for major morbidity when compared with AT. Due to the difference in prognostic significance between these two exercise variables future research should focus on developing a composite score, encompassing multiple CPET variables, particularly $\dot{V}{\text{O}}_{2\text{Peak}}$, to assess the risk profiles of a cohort of patients, considered to have significant comorbidity, and who may not have the physiological reserve to withstand complex major esophageal surgery with its inherent major associated pre‐, peri‐, and post‐operative challenges. In clinical practice a composite score may become an adjunct in the decision‐making process to determine the most efficacious modality of treatment between the clinician and patient. Finally, focused work is desirable regarding the potential impact of preoperative targeted exercise training programs, to improve and maximize patients’ CPET performance before oesophagectomy.

In conclusion, $\dot{V}{\text{O}}_{2\text{Peak}}$ was shown to be an important prognostic indicator of major morbidity after oesophagectomy. Cumulative survival was associated with the morbidity severity score, but not with CPET variables, though further follow‐up would be desirable in this regard. CPET therefore, has an important role in refining decisions regarding the optimum tailored treatment modality for patients diagnosed with EC, and also in planning appropriate postoperative care. Yet, clinical access to CPET remains limited, with the most recent literature reporting that only 32% of English hospitals have ready access to this utility (Huddart et al. 2013).

## Conflict of Interest

None declared.

## Supporting information




**Figure S1** **.** Univariable and multivariable analysis of factors associated with overall survival.

**Table S1** **.** Univariable and multivariable analysis of factors associated with overall survival.
