# Supplementary figures and images for: Cardiopulmonary fitness predicts postoperative major morbidity after esophagectomy for patients with cancer
Source: Physiol Rep. 2019 Jul 24;7(14):e14174. doi: 10.14814/phy2.14174 (PMC6656866; doi:10.14814/phy2.14174)

## Slide 1
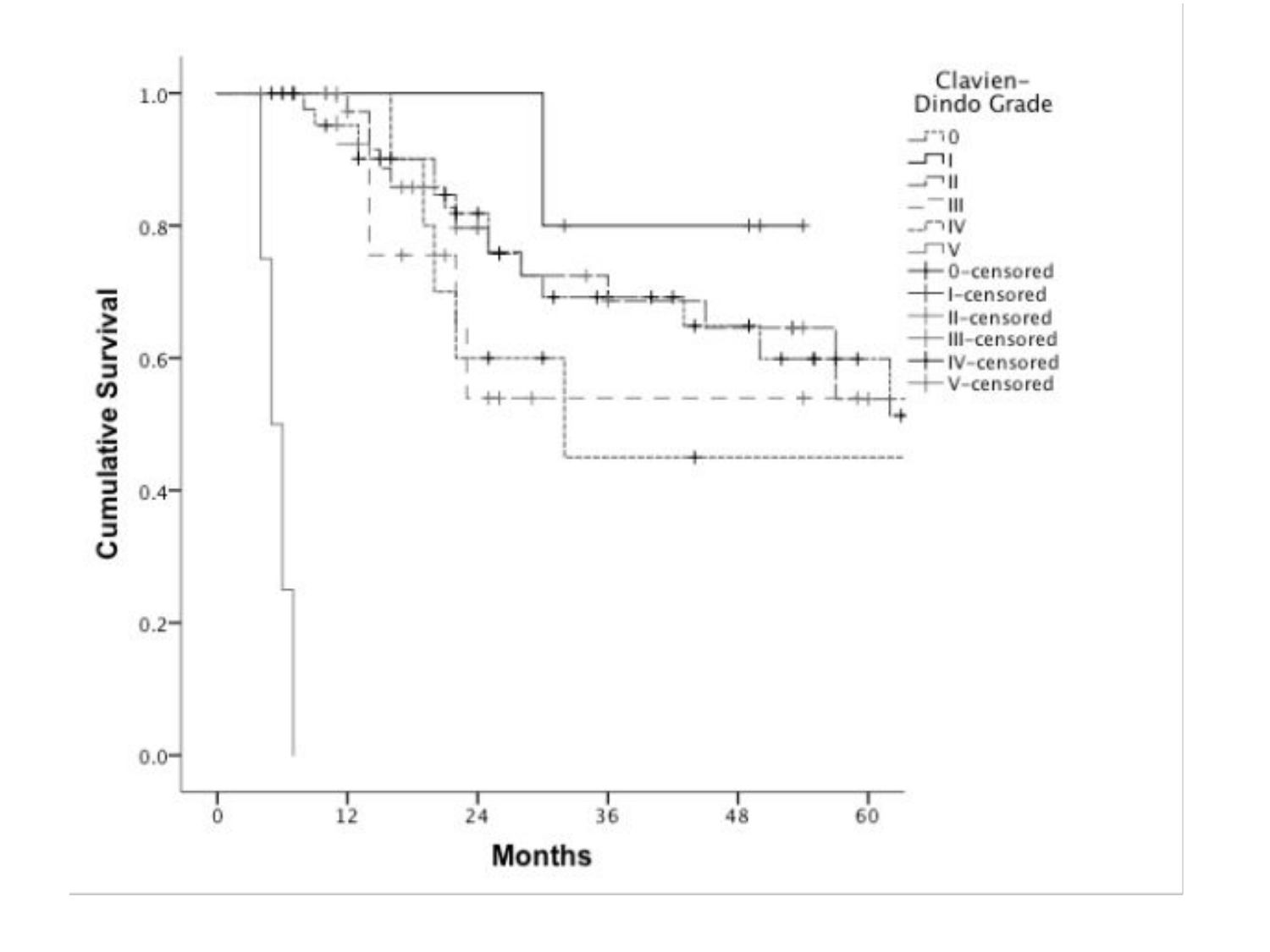

Supplement: Supplementary file 1 — Figure S1 . Univariable and multivariable analysis of factors associated with overall survival. [file PHY2-7-e14174-s001.pptx]
