# Supplementary material for: Cardiopulmonary fitness predicts postoperative major morbidity after esophagectomy for patients with cancer
Source: Physiol Rep. 2019 Jul 24;7(14):e14174. doi: 10.14814/phy2.14174 (PMC6656866; doi:10.14814/phy2.14174)
Supplement: Supplementary file 2 — Table S1 . Univariable and multivariable analysis of factors associated with overall survival. [file PHY2-7-e14174-s002.docx]

|  | Univariable  Hazard ratio (95% CI) | p-value | Multivariable  Hazard ratio (95% CI) | p-value |
| --- | --- | --- | --- | --- |
| Age (Years)  (<65 / 66-75 / >75) | 1.11 (0.64-1.19) | 0.716 |  |  |
| Gender  (Female / Male) | 1.24 (0.52-2.94) | 0.623 |  |  |
| ASA  (I / II/ III) | 1.33 (0.77-2.29) | 0.302 |  |  |
| Operative Approach  (THO / TTO) | 0.82 (0.60-1.12) | 0.210 |  |  |
| AT cut off (mL/kg/min)  (< 10.5 / >10.5) | 1.39 (0.77-2.53) | 0.279 |  |  |
| *V* O_2Peak_ cut off (mL/kg/min)  (< 17 / >17) | 0.78 (0.41-1.50) | 0.463 |  |  |
| AT (mL/kg/min) | 0.98 (0.86-1.11) | 0.737 |  |  |
| Peak v02 (mL/kg/min) | 0.95 (0.88-1.03) | 0.211 |  |  |
| Vev02 | 0.98 (0.91-1.05) | 0.537 |  |  |
| Neoadjuvant Therapy  (Yes / No) | 2.38 (1.21-4.66) | 0.012 |  | 0.862 |
| Radiological T stage  (X / 1 / 2 / 3 / 4) | 2.00 (1.35-2.99) | 0.001 |  | 0.768 |
| Radiological N stage  (0 / 1 / 2 / 3) | 1.53 (1.13-2.07) | 0.006 | 1.47 (1.02-2.13) | 0.041 |
| Pathological T stage  (X/ 1 / 2 / 3 / 4) | 2.05 (1.41-2.96) | <0.001 |  | 0.901 |
| Pathological N stage  (0 / 1 / 2 / 3) | 2.01 (1.51-2.67) | <0.001 | 1.61 (1.14-2.26) | 0.006 |
| CRM Margin  (Positive / Negative) | 3.83 (2.02-7.26) | <0.001 | 2.57 (1.27-5.19) | 0.008 |
| Calvien-Dindo Morbidity  (≤ 2 / >3) | 2.03 (1.07-3.87) | 0.031 | 2.60 (1.32-5.14) | 0.006 |

**Supplementary table 1.** Univariable and multivariable analysis of factors associated with overall survival
